# MRI-based Intra- and Peritumoral Heterogeneity in Hepatocellular Carcinoma for Microvascular Invasion Prediction and Prognostic Risk Stratification

**DOI:** 10.1148/rycan.250066

**Published:** 2025-10-24

**Authors:** Yunfei Zhang, Shutong Wang, Mingyue Song, Ruofan Sheng, Zhijun Geng, Weiguo Zhang, Mengsu Zeng

**Affiliations:** ^1^Department of Radiology, Zhongshan Hospital, Fudan University, 180 Fenglin Road, Shanghai 200032, China; ^2^Department of Radiology, Shanghai Institute of Medical Imaging, Fudan University, Shanghai, China; ^3^Center of Hepato-Pancreato-Biliary Surgery, The First Affiliated Hospital, Sun Yat-sen University, Guangzhou, China; ^4^Department of Radiology, The Fourth Affiliated Hospital of Soochow University, Medical Center of Soochow University, Suzhou, China; ^5^Department of Radiology, Sun Yat-sen University Cancer Center; State Key Laboratory of Oncology in Southern China, Guangzhou, China

**Keywords:** Liver, MRI, Oncology, Hepatocellular Carcinoma, Microvascular Invasion, Tumor habitat, Intratumoral Heterogeneity, Peritumoral Heterogeneity

## Abstract

**Purpose:**

To evaluate an MRI-based strategy for quantifying intra- and peritumoral heterogeneity (ITH and PTH) in hepatocellular carcinoma (HCC) and develop ITH- and PTH-based models for diagnosing microvascular invasion (MVI) and stratifying prognostic risk.

**Materials and Methods:**

Patients with HCC (≤5 cm) were retrospectively included from three different institutions from March 2012 to September 2023 and divided into internal training, internal testing, and external testing cohorts. Tumor and peritumoral tissues in MR images were categorized into distinct habitats using unsupervised clustering algorithms. High-throughput radiomic features were extracted from each habitat. The degree of feature variation within each habitat was quantified to derive characteristics representing ITH and PTH. Engineered features were developed to train machine learning models for MVI diagnosis. Kaplan-Meier survival curves and Cox regression analysis were used for survival analysis.

**Results:**

A total of 432 patients (mean age, 54.31 years ± 11.15 [SD]; 371 male) were included. The TH_DNN model, constructed using ITH- and PTH-based quantitative features combined with a deep neural network (DNN), demonstrated the best predictive performance for MVI across the three datasets (area under the receiver operating characteristic curve range = 0.82–0.99). The subgroup predicted as MVI positive with the TH_DNN model exhibited a poorer prognosis than the MVI-negative subgroup. In terms of overall survival and postoperative recurrence, the hazard ratios for MVI diagnosis were 2.79 (95% CI: 1.35, 5.75; *P* = .006) and 2.17 (95% CI: 1.38, 3.43; *P* < .001), respectively.

**Conclusion:**

This study developed a strategy for quantifying ITH and PTH, which was valuable for noninvasive and accurate identification of MVI and prognostic risk in patients with HCC.

**Keywords:** Liver, MRI, Oncology, Hepatocellular Carcinoma, Microvascular Invasion, Tumor habitat, Intratumoral Heterogeneity, Peritumoral Heterogeneity

*Supplemental material is available for this article.*

© The Author(s) 2025. Published by the Radiological Society of North America under a CC BY 4.0 license.

## Graphical abstract

**Figure:**
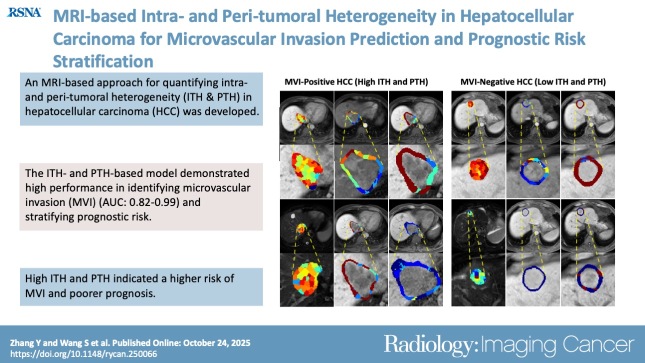

**Summary**

An MRI-based approach for quantifying intra- and peritumoral heterogeneity in hepatocellular carcinoma was developed, showing potential for accurately identifying microvascular invasion and stratifying prognostic risk.

**Key Points**

■ This multicenter study evaluated 432 patients with hepatocellular carcinoma by extracting radiomic MRI features of regions with intra- and peritumoral heterogeneity (ITH and PTH) to quantify heterogeneity.
■ By combining ITH- and PTH-derived quantitative features with a deep neural network, the model demonstrated excellent performance in identifying microvascular invasion (area under the receiver operating characteristic curve range across training and test sets, 0.82–0.99).
■ The ITH- and PTH-based model effectively stratified prognostic risk, with hazard ratios of 2.79 (*P* = .006) for overall survival and 2.17 (*P* < .001) for recurrence-free survival.

## Introduction

Hepatocellular carcinoma (HCC), a major subtype of primary liver cancer, ranks among the leading causes of cancer-related morbidity and mortality worldwide (1,2). Considerable data have shown that the presence of microvascular invasion (MVI) often indicates a poor prognosis in patients with HCC, signifying a higher likelihood of postoperative recurrence and adverse outcomes (3,4). Therefore, the ability to diagnose MVI noninvasively and accurately at an early stage could substantially enhance clinical decision-making and facilitate personalized treatment strategies for patients with HCC (5).

Increasing evidence has emphasized that intratumoral heterogeneity (ITH) offers valuable insights for identifying novel biomarkers and developing personalized therapeutic strategies (6). For instance, ITH has been preliminarily demonstrated as an imaging predictor for breast cancer and brain tumors, aiding in tumor prognosis assessment (7–9). Moreover, changes in the peritumoral microenvironment, which often mediate upstream signaling pathways involved in tumor metastasis, suggest that assessing peritumoral heterogeneity (PTH) may reveal novel diagnostic and prognostic markers (10,11). Nonetheless, research investigating ITH and PTH in HCC using medical imaging remains scarce.

The tumor habitat refers to regions composed of tissues sharing similar physiologic, metabolic, functional, or genetic characteristics. These habitats provide crucial insights into tumor components, which inherently reflect the compositional heterogeneity of the tumor (12–14). The distinct compositional information derived from tumor habitats may serve as a foundation for novel strategies to evaluate both ITH and PTH.

The objectives of this study were to develop a technical framework based on tumor and peritumoral habitats for quantifying ITH and PTH in HCC and to construct predictive models using ITH- and PTH-derived metrics for noninvasively diagnosing MVI and evaluating postoperative prognosis in patients with HCC undergoing liver resection. Specifically, the core method involves encoding the tumor and peritumoral regions into different habitats, calculating multiple radiomics features within each habitat, and quantifying the distribution heterogeneity of these features across habitats. This approach facilitates the extraction of quantitative metrics for ITH and PTH, which hold potential as imaging biomarkers for tumor diagnosis, pathologic characterization, and prognostic assessment.

## Materials and Methods

**Figure 1:**
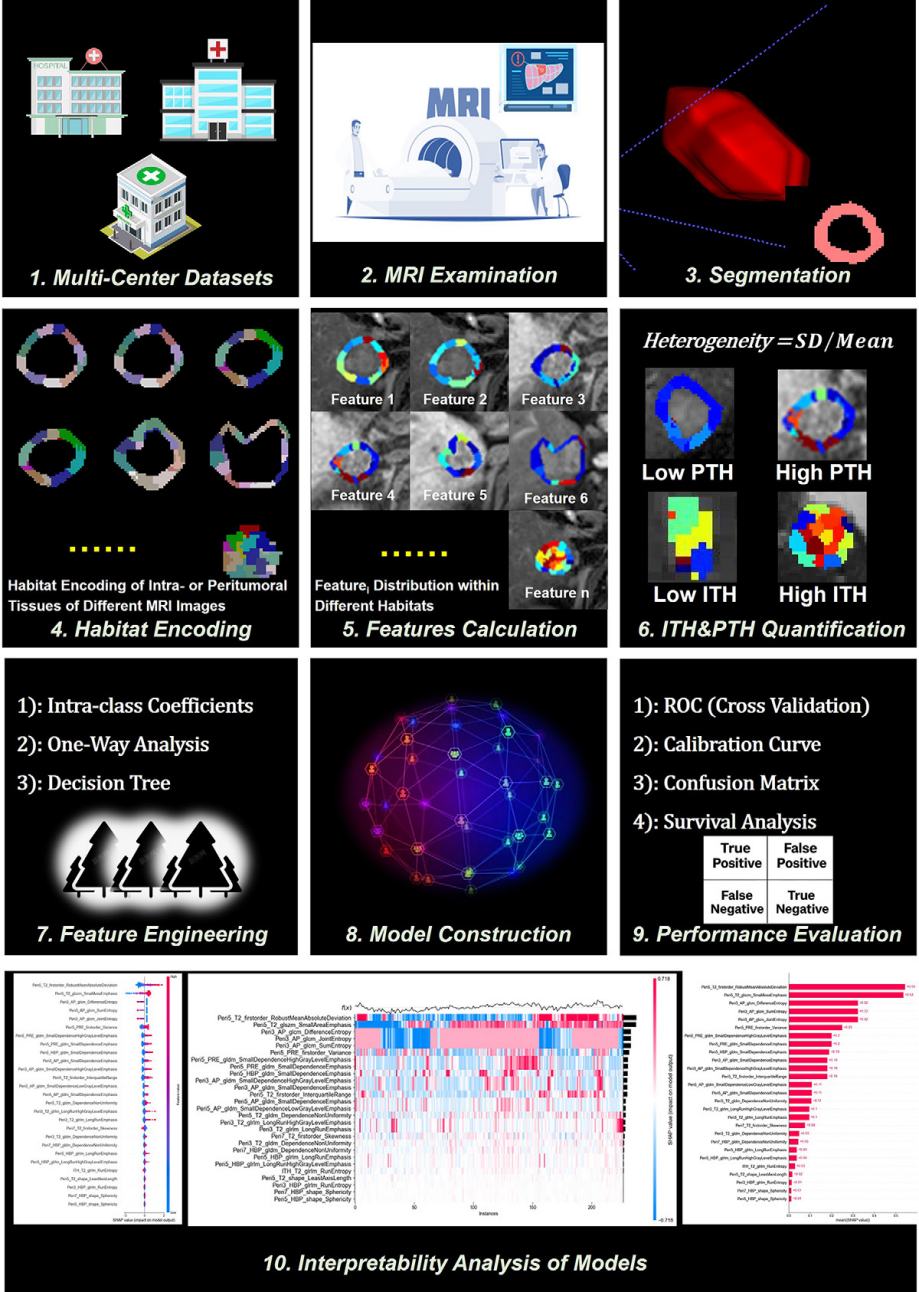
Technical flowchart of the study. The main workflow includes the integration of multicenter data, MRI data acquisition, segmentation of tumoral and peritumoral tissues, habitat encoding, feature extraction, quantification of intratumoral heterogeneity (ITH) and peritumoral heterogeneity (PTH), feature engineering, development of a diagnostic model for microvascular invasion, model evaluation and survival analysis, and interpretability analysis. Some elements are derived from the experimental results of the study, which may also be presented more comprehensively in other figures. These representations serve distinct illustrative purposes. ROC = receiver operating characteristic.

Figure 1 illustrates the overall technical workflow of this study. The detailed method is described below.

### Patients

This retrospective study received approval from the institutional ethics committees of the three participating institutions: Zhongshan Hospital, Fudan University (institution 1), The Fourth Affiliated Hospital of Soochow University (institution 2), and Sun Yat-sen University Cancer Center (institution 3). Owing to the retrospective nature of the study, the requirement for informed consent was waived. Between March 2012 and September 2023, consecutive patients with HCC who met the inclusion criteria were initially considered. Subsequently, the exclusion criteria were applied to determine the final cohort. The inclusion criteria included patients with pathologically confirmed HCC with a single nodule and patients who underwent curative hepatectomy and preoperative gadoxetate disodium–enhanced MRI scanning. The exclusion criteria included patients who underwent liver-directed therapies before surgery, such as liver transplantation, chemotherapy, radiation therapy, transarterial chemoembolization, radiofrequency ablation, or systemic therapy; inadequate MRI sequences due to scanning failure or related issues; poor MR image quality, such as severe motion artifacts or distortion; a large HCC size (diameter > 5 cm); and a time interval of more than 3 weeks between MRI and surgery. Data from institution 1 were split into an internal training and testing set using random stratified sampling (7:3 ratio). Data from institutions 2 and 3 were combined to form the external testing set.

### MRI Scanning

All included patients underwent preoperative MRI examination. MRI protocols included T2-weighted imaging, precontrast-enhanced T1-weighted imaging, diffusion-weighted imaging, and gadoxetate disodium–enhanced T1-weighted imaging at the arterial phase (AP), portal venous phase, transitional phase, and hepatobiliary phase (HBP). Four MRI sequences (T2-weighted imaging, precontrast-enhanced T1-weighted imaging, AP, and HBP) were selected for ITH and PTH assessments. Detailed scanning parameters and rationale for sequence selection when analyzing ITH and PTH are provided in Appendix S1.

### Quantification of ITH and PTH

**Image preprocessing**

MR images were resampled to a voxel size of 1.0 × 1.0 × 3.0 mm^3^ and normalized to mitigate the impact of variability in signal intensity ranges across sequences.

**Tumoral and peritumoral segmentations**

HCC margins across all sequences were independently delineated by six observers to ensure complete coverage of the tumor region: internal dataset (Y.Z. and R.S., with 8 and over 10 years of experience in abdominal MRI, respectively); external institution 2 dataset (M.S. and W.Z., with 3 and 15 years of experience in abdominal MRI, respectively); and external institution 3 dataset (Z.G. and S.W., with 10 and 12 years of experience in abdominal MRI, respectively). Based on the HCC segmentation, peritumoral tissues at distances of 3, 5, and 7 mm from the tumor boundary (designated as Peri3, Peri5, and Peri7, respectively) were segmented using dilation algorithms implemented in the Python-based package SciPy. More details are provided in Appendix S1.

**Intratumoral and peritumoral habitat encoding**

The simple linear iterative clustering algorithm (an unsupervised clustering method) was applied to independently encode the intratumoral region and each peritumoral region at different scales (Peri3, Peri5, and Peri7) into 50 habitats each, based on similarities in intensity, texture, and spatial structure. Detailed technical considerations for encoding these regions into their respective 50 habitats are provided in Appendix S1.

**Feature extraction**

Radiomic features were extracted for each habitat in the intratumoral and peritumoral regions using the Python-based package PyRadiomics (15). To enable comparative analysis, radiomic features were also extracted for the entire HCC region (without habitat segmentation). For each sequence (T2-weighted imaging, precontrast-enhanced T1-weighted imaging, AP, and HBP), 107 features were extracted, yielding 428 features (107 × 4 sequences) per habitat or entire HCC region. The extracted features are detailed in Table S1.

**Quantification of heterogeneity**

The coefficient of variation is widely used to characterize the variability of numerical values across different instances—a value of 0 indicates complete homogeneity, whereas higher values represent increasing heterogeneity in the distribution. Therefore, heterogeneity was quantified using the coefficient of variation for each radiomic feature across habitats, reflecting the uneven distributions of features across all habitats:


$$heterogeneity\;parameter_{i}=\frac{standard\;deviation_{i}}{mean_{i}},$$

where SD_i_ and mean_i_ refer to the SD and mean of feature_i_ across various habitats, respectively. This parameter reflects the uneven distribution of feature_i_ across habitats, with higher values indicating greater distribution heterogeneities. We derived 1712 heterogeneity features (428 features × four regions [intratumoral, Peri3, Peri5, and Peri7]) to quantify ITH and PTH.

The source codes for image preprocessing, peritumoral segmentation, feature extraction, and heterogeneity quantification are publicly available in open-source format at *https://github.com/yunfei920406/ITH_PTH_HCC_Project*.

### Clinical Characteristics and Prognostic Follow-up

Clinical data, including the pathologic diagnoses of MVI, were extracted from the electronic medical record systems of the participating hospitals. MVI was defined as the presence of tumor cell nests in the portal vein, hepatic vein, and tumor capsular vessel lined by endothelium (16). The tumor diameter was measured as the longest axis of the lesion. Patients from institution 1 underwent regular follow-up after surgery at 1- to 4-month intervals. Detailed survival data were collected, including surgery dates and information related to recurrence, metastasis, and mortality. The follow-up data for most patients from institution 2 and institution 3 were incomplete—primarily due to loss to follow-up and other factors—and were therefore excluded from subsequent survival analysis.

### Models Construction

Feature selection was carried out in three steps. First, features with an intraobserver or interobserver intraclass correlation coefficient of less than 0.800 were excluded. Second, features without evidence of a difference (Student *t* test or Mann-Whitney *U* test) between the MVI-negative and MVI-positive groups were excluded. Third, a decision tree algorithm was used to select the top 20 features based on their importance scores, calculated from each feature’s contribution to reducing model impurity. Using a feature subset based on ITH and PTH (ie, the tumor heterogeneity [TH] feature subset), seven models were developed by combining the TH feature set with a logistic regression, random forest, decision tree, support vector machine, AdaBoost, k-nearest neighbors, or deep neural network (DNN) algorithm. Using the same three-step feature-selection method described above, radiomic features of HCC (without habitat encoding) were selected to form a feature subset (named “Rad”). This feature subset was combined with the aforementioned seven algorithms to construct seven distinct predictive models. Open-source codes for feature engineering and model construction are available at *https://github.com/yunfei920406/ITH_PTH_HCC_Project*.

### Model Evaluation

The diagnostic performance of our models was evaluated via confusion matrix analysis and receiver operating characteristic (ROC) curve analysis. The following metrics were calculated: area under the ROC curve (AUC), sensitivity, specificity, accuracy, precision, recall, and F1 score. Calibration errors, derived from calibration curve analysis, were also used to evaluate predictions. Our models were developed based on an internal training dataset and evaluated on independent internal testing and external testing cohorts.

### Statistical Analysis

Before conducting this study, we used power analysis based on ROC analysis to estimate the required sample size, setting the statistical power and significance level to 0.90 and .05, respectively. We analyzed the stability of the selected ITH- and PTH-derived features (TH feature subset) computed with varying habitat settings (*n* = 30, 40, 50, 60, or 70), with intraclass correlation coefficients. Univariable and multivariable logistic regression models were used to assess relationships between clinical variables, model prediction probabilities, and the MVI status. Kaplan-Meier survival curves and the log-rank test were applied to analyze the relationship between postoperative recurrence, overall survival (OS), and MVI subgroups. The restricted mean survival time (RMST) and restricted mean recurrence-free time (RMRFT) were calculated. The RMST and RMRFT reflect the average survival time and average recurrence-free survival (RFS) time of patients within a specific time window by calculating the area under the OS and RFS curves for that period. Cox regression analysis was conducted to identify prognostic risk factors. A two-tailed *P* < .05 was considered statistically significant. Survival analysis was based on the internal training and testing datasets. Shapley additive explanations (SHAP) analysis was conducted for interpretability analysis. SHAP analysis was performed using the Python-based package shap (17). A SHAP model was established using the internal training data as background data. SHAP values were independently calculated for the internal and external testing datasets, after which they were used for standalone interpretability analysis. Power analysis was conducted using PASS software (version 2021; NCSS). Other statistical analyses were performed using R software (version 4.1.0; R Foundation for Statistical Computing).

## Results

### Patient Characteristics

**Figure 2:**
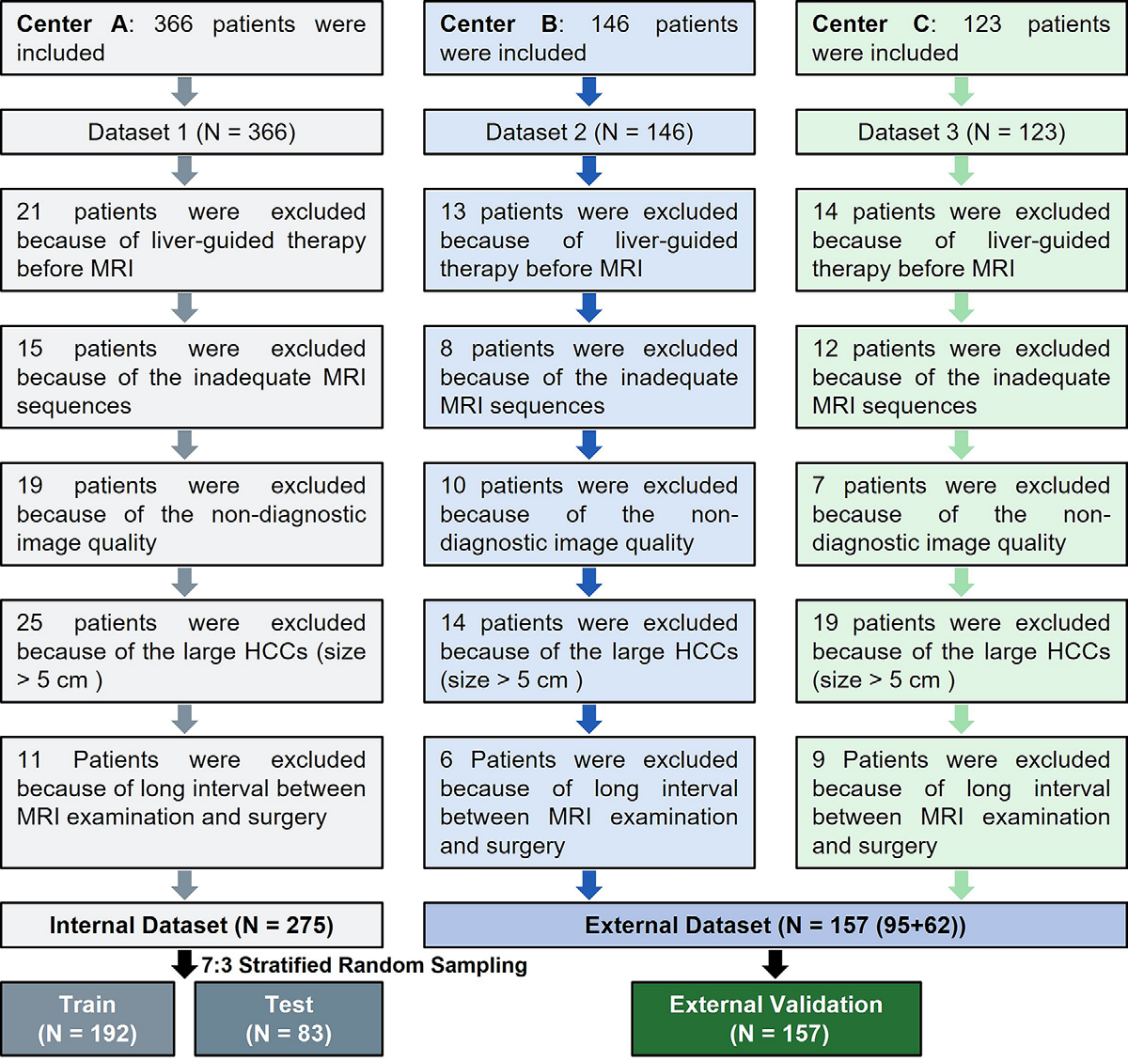
Flow diagram of patient inclusion and exclusion. Center A, B, and C, is institution 1, 2, and 3, respectively. HCC = hepatocellular carcinoma.

Initially, 366 patients from institution 1 were included, after which 91 were excluded, resulting in 275 patients who were ultimately enrolled. From institution 2, 146 patients were initially included, with 51 excluded, leaving 95 patients. Similarly, 123 patients from institution 3 were included initially, with 61 excluded, resulting in 62 patients (Fig 2).

Data from institution 1 were divided into an internal training cohort (*n* = 192; 169 [88.0%] male, 23 [12.0%] female; mean age, 52.77 years ± 11.26 [SD]; tumor size, 2.09 cm ± 0.99; 45 [23.4%] patients diagnosed with MVI) and an internal testing cohort (*n* = 83; 70 [84.3%] male, 13 [15.7%] female; mean age, 53.13 years ± 11.15; tumor size, 2.10 cm ± 1.04; 20 [24.1%] patients diagnosed with MVI), using stratified sampling. Data from institutions 2 and 3 were combined to form the external testing cohort (*n* = 157; 132 [84.1%] male, 25 [15.9%] female; mean age, 56.83 years ± 10.56; tumor size, 2.98 cm ± 1.05; 53 [33.8%] patients diagnosed with MVI). Detailed patient characteristics are shown in Table 1.

**Table 1:** Clinical Characteristics of the Internal Training, Internal Testing, and External Testing Datasets

Characteristic	Internal Training (*n* = 192)	Internal Testing (*n* = 83)	External Testing (*n* = 157)
Age (y)	52.77 ± 11.26 (24–81)	53.13 ± 11.15 (23–80)	56.83 ± 10.56 (25–81)
Sex			
Male	88.0 (169/192)	84.3 (70/83)	84.1 (132/157)
Female	12.0 (23/192)	15.7 (13/83)	15.9 (25/157)
HBV	80.2 (154/192)	71.1 (59/83)	98.1 (154/157)
HCV	1.0 (2/192)	3.6 (3/83)	1.9 (3/157)
Cirrhosis			
With	85.4 (164/192)	78.3 (65/83)	94.9 (149/157)
Without	14.6 (28/192)	21.7 (18/83)	5.1 (8/157)
Tumor diameter (cm)	2.09 ± 0.99 (0.5–5.0)	2.10 ± 1.04 (0.6–5.0)	2.98 ± 1.05 (1.0–5.0)
Differentiation			
Well to moderate	64.1 (123/192)	54.2 (45/83)	70.7 (111/157)
Poor	35.9 (69/192)	45.7 (38/83)	29.3 (46/157)
MVI			
Negative	76.6 (147/192)	75.9 (63/83)	66.2 (104/157)
Positive	23.4 (45/192)	24.1 (20/83)	33.8 (53/157)

Note.—Data are reported as means ± sds with ranges in parentheses for continuous variables and as percentages with absolute numbers in parentheses for categorical variables. HBV = hepatitis B virus, HCV = hepatitis C virus, MVI = microvascular invasion.

### Visualization and Quantification of ITH and PTH

**Figure 3:**
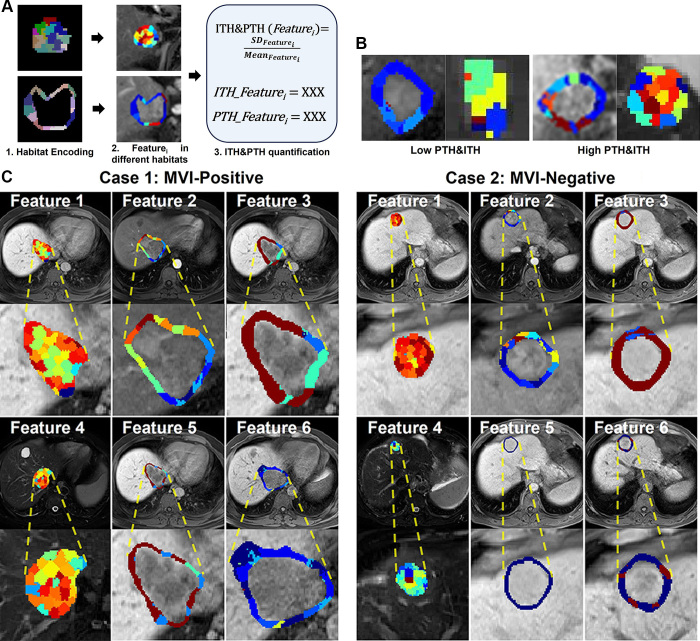
Intratumoral heterogeneity (ITH) and peritumoral heterogeneity (PTH) visualization and quantification. **(A)** The core framework of quantifying the ITH and PTH. First, the PTH and ITH tissues are encoded into different habitats with unsupervised clustering algorithms (each color represents a different habitat). Second, radiomics feature_i_ was calculated in each habitat (the colors represent the values of feature_i_ in different habitats). Third, the heterogeneity of the feature_i_ distribution across habitats is quantified using (CV=SDFeatureiMeanFeaturei). *SD_Feature_i__* and *Mean_Feature_i__* refer to the SD and mean of feature_i_ across various habitats, respectively. Thus, the heterogeneity of the feature_i_ distribution across these habitats was quantified, enabling the derivation of feature_i_ representing ITH and PTH (ITH & PTH(Feature_i_)). As high-throughput radiomics features can be calculated in each habitat, by extending this method to all features, we can derive a comprehensive set of quantitative representations of ITH and PTH. **(B)** Schematic representation of low versus high ITH and PTH. **(C)** Visualization of ITH and PTH for one patient who was microvascular invasion (MVI) positive (50-year-old male) and one patient who was MVI negative (71-year-old male), derived from the heterogeneity distribution of various radiomics features across ITH and PTH habitats. The patient who was MVI positive exhibited higher ITH and PTH compared with the patient who was MVI negative, as evidenced by greater heterogeneity in feature distributions across habitats. The color maps, representing feature values within distinct habitats, are overlaid on the MR images.

Figure 3A illustrates the core concept of the heterogeneity-quantification framework developed in this study. Tumoral and peritumoral tissues were segmented into distinct habitats, and high-throughput radiomics features were extracted from each habitat. The heterogeneity of each radiomic feature’s distribution across these habitats was then quantified, enabling the derivation of features representing ITH and PTH. Figure 3B presents examples of high and low ITH and PTH. Figure 3C shows the visualization of heterogeneity for one patient who was MVI positive and one patient who was MVI negative. Our results indicate that the patient who was MVI positive exhibited significantly greater ITH and PTH, as evidenced by more heterogeneous feature distributions across habitats, compared with those of the patient who was MVI negative.

### Model Construction and Evaluation

A three-step feature selection process—comprising intraclass correlation coefficient filtering, univariate testing, and decision tree–based ranking—was conducted. This procedure yielded 269, 130, and 20 retained features, respectively, for the conventional radiomics-based subset (Rad), and 1424, 136, and 20 retained features, respectively, for the ITH- and PTH-derived subset (TH feature subset) (Tables S2, S3). Tables 2 and S4 summarize the diagnostic performance of models that identified MVI using ITH- and PTH-based features, alongside conventional radiomics features. The DNN model incorporating the features of the TH subset (TH_DNN model) demonstrated superior diagnostic performance across all datasets, when compared with models using other feature subsets. The diagnostic metrics for the TH_DNN model were as follows: internal training set: AUC = 0.99, sensitivity = 100.0%, specificity = 96.6%, accuracy = 97.4%, precision = 90.0%, recall = 100.0%, F1 score = 0.95; internal testing set: AUC = 0.88, sensitivity = 90.0%, specificity = 73.0%, accuracy = 77.1%, precision = 51.4%, recall = 90.0%, F1 score = 0.66; external testing set: AUC = 0.82, sensitivity = 66.0%, specificity = 81.7%, accuracy = 76.4%, precision = 64.8%, recall = 66.0%, F1 score = 0.65. The TH_DNN model consistently outperformed conventional radiomics models in both the internal and external testing cohorts.

**Table 2:** Diagnostic Performance of Models Based on ITH and PTH

Model and Group	AUC	Sens (%)	Spe (%)	ACC (%)	Prec (%)	Recall (%)	F1 Score
TH_SVM							
Internal training	0.97	97.8	92.5	93.8	80.0	97.8	0.88
Internal testing	0.76	95.0	50.8	61.5	38.0	95.0	0.54
External testing	0.63	56.6	68.3	64.3	47.6	56.6	0.52
TH_RF							
Internal training	0.96	95.6	89.1	90.6	72.9	95.6	0.83
Internal testing	0.79	100.0	60.3	69.9	44.4	100.0	0.61
External testing	0.64	83.0	40.4	54.8	41.5	83.0	0.55
TH_LG							
Internal training	>0.99	100.0	100.0	100.0	100.0	100.0	1.00
Internal testing	0.79	90.0	63.5	69.9	43.9	90.0	0.59
External testing	0.67	64.2	72.1	69.4	54.0	64.2	0.59
TH_KNN							
Internal training	0.83	66.7	80.3	77.1	50.9	66.7	0.58
Internal testing	0.74	70.0	76.2	74.7	48.3	70.0	0.57
External testing	0.62	75.5	42.3	53.5	40.0	75.5	0.52
TH_Ada							
Internal training	>0.99	100.0	100.0	100.0	100.0	100.0	1.00
Internal testing	0.74	60.0	81.0	75.9	50.0	60.0	0.55
External testing	0.65	47.2	76.9	66.9	51.0	47.2	0.49
TH_DT							
Internal training	0.99	100.0	93.2	94.8	81.8	100.0	0.90
Internal testing	0.71	60.0	87.3	80.7	60.0	60.0	0.60
External testing	0.52	34.0	73.1	59.9	39.1	34.0	0.36
TH_DNN							
Internal training	0.99	100.0	96.6	97.4	90.0	100.0	0.95
Internal testing	0.88	90.0	73.0	77.1	51.4	90.0	0.66
External testing	0.82	66.0	81.7	76.4	64.8	66.0	0.65

Note.—Sensitivity (Sens), specificity (Spe), accuracy (ACC), precision (Prec), and recall are percentages. These models were constructed using the feature subset derived from the quantitative characterization of intratumoral heterogeneity (ITH) and peritumoral heterogeneity (PTH). The feature subset was referred to as TH. Various predictive algorithms were used, including logistic regression (LG), random forest (RF), decision tree (DT), support vector machine (SVM), AdaBoost (Ada), k-nearest neighbors (KNN), and deep neural network (DNN). The diagnostic performance was evaluated using multiple metrics, including area under the receiver operating characteristic curve (AUC), sensitivity, specificity, accuracy, precision, recall, and F1 score.

**Figure 4:**
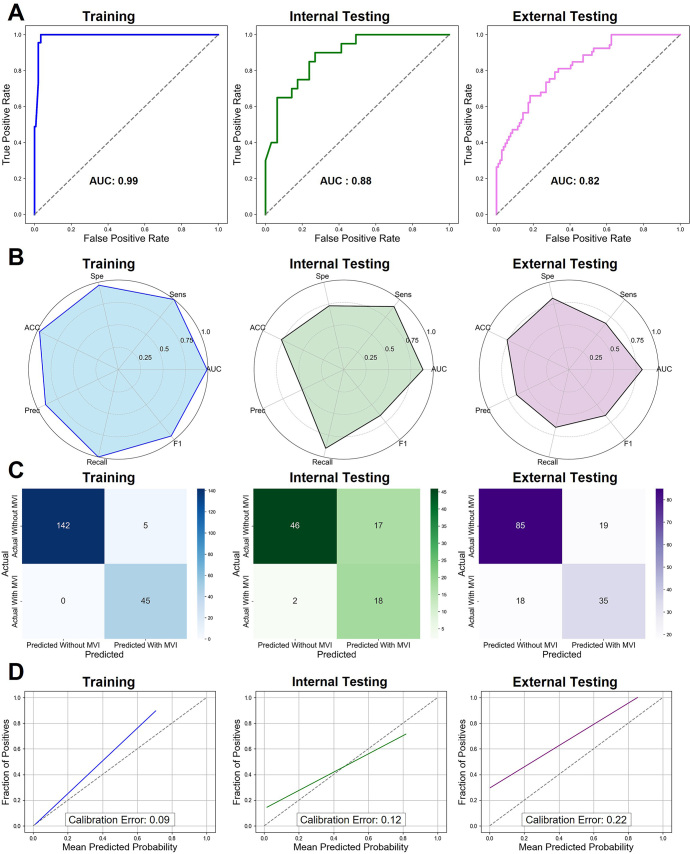
Diagnostic performance evaluation of the TH_DNN model across the internal training, internal testing, and external testing cohorts. **(A)** Receiver operating characteristic curves illustrate the diagnostic accuracy of the TH_DNN model in the training (blue), testing (green), and external testing (pink) cohorts. **(B)** Radar plots compare key performance metrics—accuracy (ACC), sensitivity (Sens), specificity (Spe), precision (Prec), recall, F1 score, and area under the receiver operating characteristic curve (AUC)—across the three cohorts. **(C)** Confusion matrices display the classification results for each cohort. **(D)** Calibration curves assess the agreement between predicted probabilities and actual microvascular invasion (MVI) status, with calibration errors of 0.09, 0.12, and 0.22 for the internal training, internal testing, and external testing cohorts, respectively. DNN = deep neural network, TH = tumor heterogeneity.

Figure 4 illustrates the ROC curves, confusion matrices, and calibration curve analyses, with calibration errors of 0.09, 0.12, and 0.22 for the internal training, internal testing, and external testing datasets, respectively. As shown in Table S5, univariable and multivariable logistic regression analyses, including TH_DNN-predicted probabilities and other clinical indicators, revealed that only the TH_DNN-predicted probabilities retained statistical significance (*P* < .001). Consequently, clinical variables were not used to construct a combined model with the TH_DNN model.

Additionally, we performed univariable regression to verify whether the model’s diagnostic predictions were independent of MRI field strength. As shown in Table S6, the model’s predictions were not affected by the field strength (*P* = .49). As shown in Figure S2, the heterogeneity metrics remained relatively stable across different habitat settings, with all intraclass correlation coefficients exceeding 0.7.

### Model Interpretability Analysis

**Figure 5:**
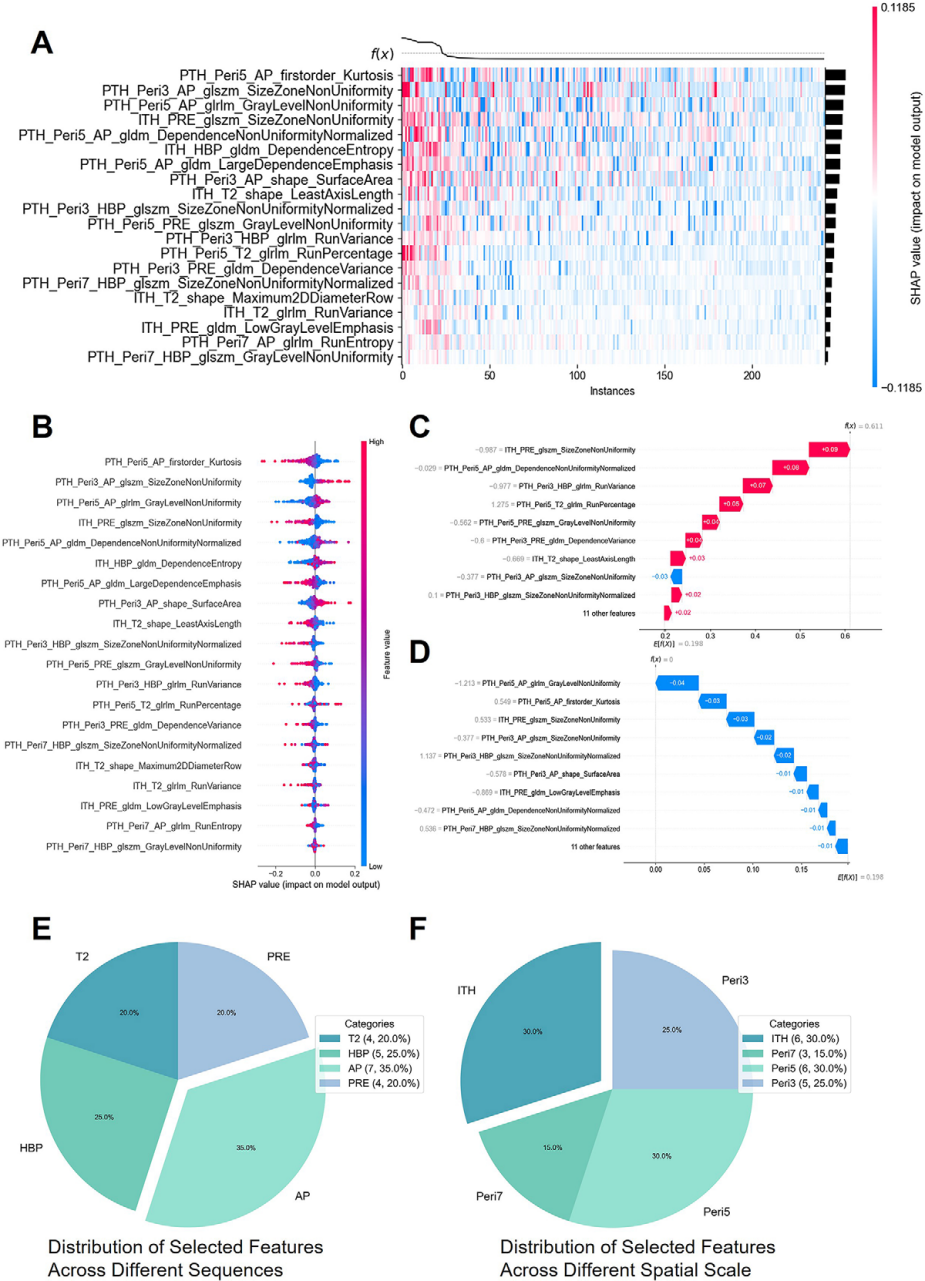
Interpretability analysis of the TH_DNN model using Shapley additive explanations (SHAP). **(A)** The heat map visualizes the SHAP values of the top-ranked features for individual predictions in the TH_DNN model. Each row represents a feature, and each dot represents a SHAP value for a single instance. Positive SHAP values (red) indicate a positive contribution to the model’s prediction, while negative SHAP values (blue) indicate a negative contribution. **(B)** The SHAP dependence plot highlights the relationship between feature values and SHAP values for the most impactful features, illustrating how variations in feature values influence model predictions. **(C)** The SHAP force plot visualizes the feature contributions for a representative patient who was microvascular invasion positive (52-year-old male). **(D)** The SHAP force plot highlights the feature contributions for a representative patient who was microvascular invasion negative (58-year-old male). The distribution of selected features of the tumor heterogeneity (TH) subset (*n* = 20) across different **(E)** MRI sequences and **(F)** spatial scales. AP = gadoxetate disodium–enhanced T1-weighted imaging at the arterial phase, DNN = deep neural network, HBP = gadoxetate disodium–enhanced T1-weighted imaging at the hepatobiliary phase, ITH = intratumoral heterogeneity, Peri3 = peritumoral tissue at distance of 3 mm, Peri5 = peritumoral tissue at distance of 5 mm, Peri7 = peritumoral tissue at distance of 7 mm, PRE = precontrast-enhanced T1-weighted imaging, T2 = T2-weighted imaging.

Because the TH_DNN model demonstrated the best predictive performance, SHAP analysis was conducted to investigate its decision-making mechanism. Figure 5A displays a SHAP value heat map, elucidating the impact of different features on the model output for individual patients. The SHAP importance rankings are provided in Table S7. Notably, the PTH_Peri5_AP_firstorder_Kurtosis, PTH_Peri3_AP_glszm_SizeZoneNonUniformity, and PTH_Peri5_AP_glrlm_GrayLevelNonUniformity features were the top contributors, with distinct patterns differentiating patients who were MVI positive and MVI negative. Figure 5B summarizes the global importance of different features, emphasizing their association with MVI diagnosis. The high SHAP value variability observed for the PTH_Peri5_AP_firstorder_Kurtosis feature underscored its utility in MVI identification. Representative predictions for patients who are MVI negative (Fig 5C) and MVI positive (Fig 5D) further illustrate the decision-making process, showing how specific features influenced the confidence in the model in supporting an MVI diagnosis. We also analyzed the distribution of selected features from the TH subset (*n* = 20) across different MRI sequences and spatial scales (Fig 5E, 5F). Specifically, we derived seven features from AP imaging, five from HBP imaging, four from T2-weighted imaging, and four from precontrast-enhanced T1-weighted imaging. In terms of spatial distribution, we derived six features from both Peri5 and ITH, five from Peri3, and three from Peri7.

### Prognostic Evaluation and Risk Stratification

**Figure 6:**
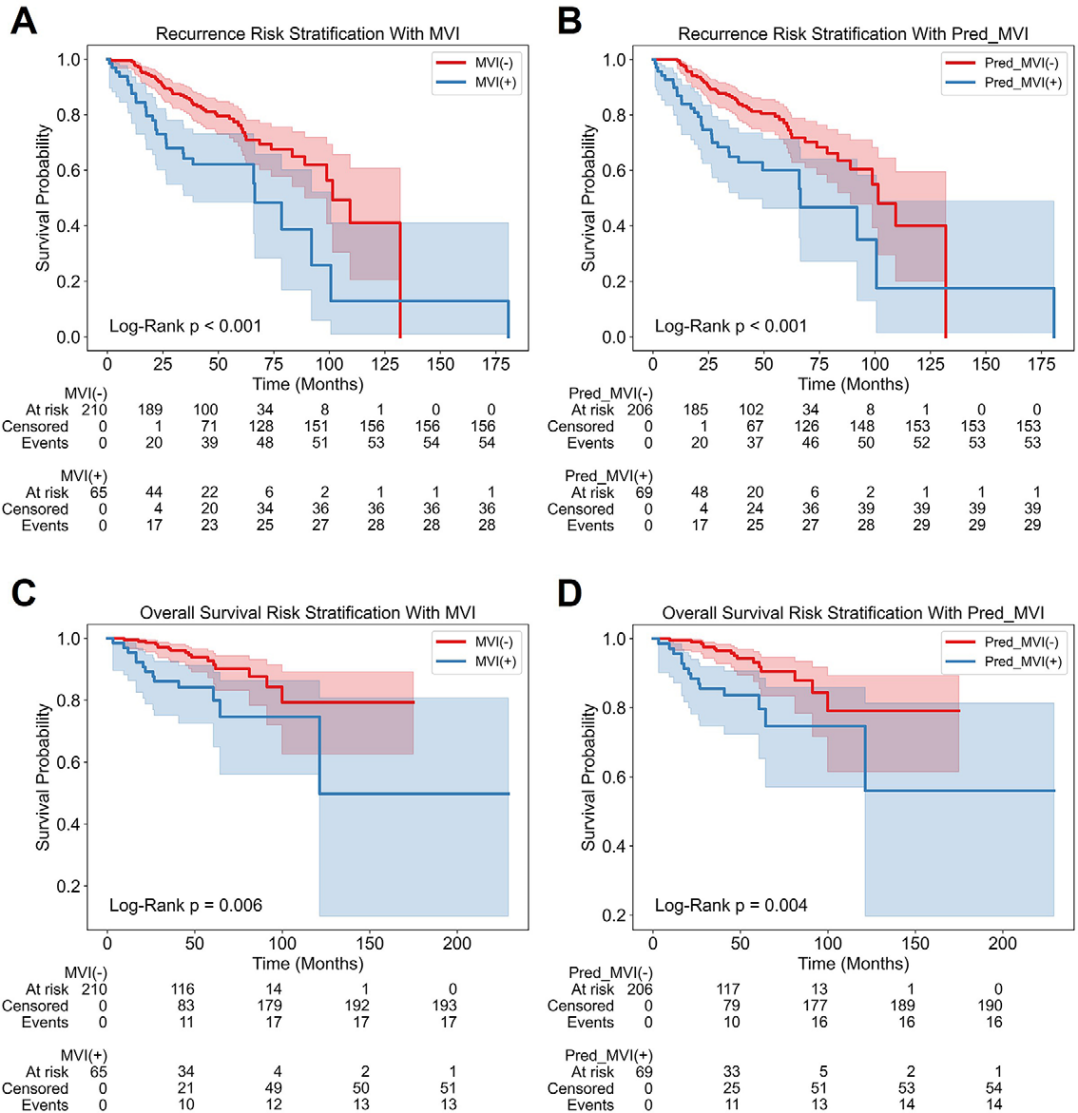
Kaplan-Meier curves for recurrence-free survival and overall survival. **(A)** Kaplan-Meier survival curve for recurrence-free survival between pathologically conformed microvascular invasion positive (MVI(+)) and negative (MVI(-)). **(B)** Kaplan-Meier survival curve for recurrence-free survival between predicted MVI positive (Pred_MVI(+)) and negative (Pred_MVI(-)). **(C)** Kaplan-Meier survival curve for overall survival between MVI positive and MVI negative. **(D)** Kaplan-Meier survival curve for overall survival between predicted MVI positive and predicted MVI negative.

The study population was divided into separate groups based on pathologic MVI diagnosis—the MVI-positive and MVI-negative groups and TH_DNN model predictions (ie, the Pred_MVI-positive and Pred_MVI-negative groups). Significant differences in Kaplan-Meier survival curves for RFS and OS were observed between the MVI-positive and MVI-negative groups (log-rank *P* < .001 and *P* = .006, respectively) and the Pred_MVI-positive and Pred_MVI-negative groups (log-rank *P* < .001 and *P* = .004, respectively) (Fig 6). As shown in Table 3, the MVI-positive group had a worse prognosis than the MVI-negative group: median RFS (71.7 vs 94.0 months; *P* < .001), 3-year RMRFT (28.0 vs 33.6 months; *P* < .001), 5-year RMRFT (43.9 vs 52.5 months; *P* < .001), 3-year RMST (32.5 vs 35.0 months; *P* = .10), and 5-year RMST (53.3 vs 57.1 months; *P* = .01). Similarly, the Pred_MVI-positive group exhibited a worse prognosis than the Pred_MVI-negative group: median RFS (77.0 vs 93.7 months; *P* < .001), 3-year RMRFT (28.1 vs 33.7 months; *P* < .001), 5-year RMRFT (43.9 vs 52.7 months; *P* < .001), 3-year RMST (31.2 vs 35.1 months; *P* = .01), and 5-year RMST (53.1 vs 57.3 months; *P* = .007). Both MVI and predicted MVI were significantly associated with a worse OS and postoperative recurrence (Table S8). For OS, the hazard ratios of actual MVI and predicted MVI were 2.68 (95% CI: 1.29, 5.54; *P* = .008) and 2.79 (95% CI: 1.35, 5.75; *P* = .006), respectively. For recurrence, the hazard ratios of actual MVI and predicted MVI were 2.21 (95% CI: 1.40, 3.50; *P* < .001) and 2.17 (95% CI: 1.38, 3.43; *P* < .001), respectively.

**Table 3:** Survival Outcomes Stratified by Pathologic and Predicted MVI Status

Indexes	MVI Positive	MVI Negative	*P* Value	Pred_MVI Positive	Pred_MVI Negative	*P* Value
Median RFS	71.7 (47.9–95.6)	94.0 (85.1–102.9)	<.001	77.0 (49.9–104.1)	93.7 (84.8–102.6)	<.001
Median OS	121.3 (121.3–121.3)	NA	NA	NA	NA	NA
RMRFT (3 y)	28.0 (25.8–30.2)	33.6 (32.3–34.9)	<.001	28.1 (26.0–30.2)	33.7 (33.0–34.4)	<.001
RMRFT (5 y)	43.9 (41.5–46.4)	52.5 (51.2–53.8)	<.001	43.9 (41.7–46.2)	52.7 (51.9–53.4)	<.001
RMST (3 y)	32.5 (30.0–35.1)	35.0 (33.5–36.0)	.10	31.2 (28.8–33.6)	35.1 (33.2–36.0)	.01
RMST (5 y)	53.3 (50.8–55.9)	57.1 (55.6–58.6)	.01	53.1 (50.7–55.5)	57.3 (55.4–59.2)	.007

Note.—Data in parentheses are 95% CIs. The table presents the survival outcomes for patients stratified by pathologic microvascular invasion (MVI) status (MVI positive vs MVI negative) and predicted MVI status (Pred_MVI positive vs Pred_MVI negative). Statistical significance (*P* values) was determined for comparisons between groups. Median overall survival (OS) could not be calculated as more than 50% of patients remained alive at the last follow-up. The time unit is measured in months. The identical median OS and 95% CI values in the MVI-positive group reflect a plateau in the survival curve due to limited events. NA = not available, RFS = recurrence-free survival, RMRFT = restricted mean recurrence-free time, RMST = restricted mean survival time.

### Power Analysis

Before conducting the study, our power analysis estimated that a sample size of 57 would be necessary to achieve a statistical effect size (AUC) of 0.80, with a statistical power of 0.90 and a significance level of .05. The required sample size decreased with an increasing AUC. The TH_DNN model achieved AUC values exceeding 0.80 across all subgroups, and the internal training, internal testing, and external testing groups had sample sizes of 192, 83, and 157, respectively; thus, the study achieved a statistical power of over 0.90 for ROC analysis. A summary of the exact inputs and outputs used for power analysis are shown in Appendix S2.

## Discussion

Growing evidence has highlighted the clinical value of quantifying TH. Despite existing advances, several gaps remain: limited studies have characterized ITH and PTH in HCC, traditional radiomics techniques lack intuitive quantification and interpretability, and model generalizability and interpretability are rarely evaluated using multicenter data. In this study, we proposed a novel strategy to assess ITH and PTH using high-throughput quantitative features. A diagnostic model for preoperative prediction of MVI was developed and interpreted via SHAP analysis. Based on multicenter data, the model achieved excellent performance (AUC = 0.82–0.99) and effectively stratified postoperative prognostic risk. This approach shows promise as an innovative imaging tool to support clinical decision-making.

Several recent studies have made attempts to evaluate the feasibility of using ITH for tumor assessment. For example, Shi et al (8) proposed a pretreatment MRI-based method to quantify ITH in breast cancer, achieving an AUC range of 0.83–0.90 for predicting a pathologic complete response. Park et al (18) presented data revealing that spatiotemporal heterogeneity was associated with patient outcomes in isocitrate dehydrogenase–wild-type glioblastoma. In other studies, preliminary qualitative assessments of ITH were conducted based on traditional radiomics parameters (19–21). For example, Mu et al (19) calculated the radiomic parameters of different subregions within tumors to indirectly assess ITH. We encoded 50 distinct habitats across regions to quantify ITH and PTH. This choice reflects a trade-off between encoding complexity and the granularity of feature extraction. We acknowledge that such a setting may not be universally optimal; future studies may build on our approach and tailor the habitat number based on lesion type, size, and imaging characteristics to more effectively capture ITH and PTH. This study demonstrates that the TH_DNN model yielded the highest diagnostic efficacy among all predictive models generated, including the conventional radiomics model. This finding has a few potential explanations. First, MVI is essentially a pathologic feature of the peritumoral region, and the TH_DNN model was constructed based on ITH and PTH. Quantitative parameters derived from PTH, which originates from the peritumoral region, are more conducive to diagnosing MVI. Previous findings have also highlighted the importance of peritumoral MRI in assessing the MVI (22,23). Second, this study used multiple MRI sequences and high-throughput features calculated from different scales of peritumoral and intratumoral tissues, which provided a foundation for model construction. Third, as a deep learning model, DNN can capture complex nonlinear relationships between input features. Using backpropagation and large-scale parameter optimization algorithms, DNN is capable of more comprehensive training on multidimensional features than other approaches, enabling the identification of better decision boundaries (24,25).

SHAP-based interpretability analysis helps identify key features, and when combined with visualization results, it can further provide novel diagnostic insights. Among the 20 features in the TH feature subset, only six were derived from ITH, and the remaining 14 features originated from PTH. SHAP analysis of the TH_DNN model’s interpretability revealed that the top three most important features influencing the model’s decision were PTH_Peri5_AP_firstorder_Kurtosis, PTH_Peri3_AP_glszm_SizeZoneNonUniformity, and PTH_Peri5_AP_glrlm_GrayLevelNonUniformity. All were extracted from the peritumoral regions, underscoring the value of PTH. Notably, PTH_Peri5_AP_firstorder_Kurtosis exhibited the largest overall impact on the model’s output, suggesting that intensity distribution characteristics in the peritumoral region are particularly relevant to the diagnostic prediction. Among the top three most important features, all (100.0%) were derived from PTH, and 70.0% of the top ten were PTH derived. The above findings further support the value of PTH in MVI diagnosis. Additionally, features derived from AP (seven of 20; 35%) and HBP (five of 20; 25%) were the most prevalent in the selected features, indicative of a stronger association between AP and HBP imaging features and MVI. Previous results also identified AP- and HBP-derived image features, such as arterial hyperenhancement and hepatobiliary hypointensity, as risk factors for MVI (5,22,26).

MVI is widely regarded as an important prognostic factor in HCC. A meta-analysis involving 14 studies with 3033 patients showed that the OS of the MVI-positive group was significantly worse than that of the MVI-negative group (hazard ratio = 2.39; 95% CI: 2.02, 2.84; *P* < .001). Subgroup analysis demonstrated that MVI negatively impacted the long-term OS and RFS of patients with solitary small HCC tumors, measuring up to 2, 3, or 5 cm in diameter (27). Considering that hepatectomy is the preferred treatment of early-stage HCC, we included only HCCs with diameters of 5 cm or less, similar to that in previous research (28,29). Our results also confirmed that MVI was associated with a poor prognosis in HCC. Using the TH_DNN model, MVI (which previously required pathologic confirmation) could be diagnosed noninvasively and accurately. By categorizing patients into predicted positive and negative groups, we found that the Pred_MVI-positive group had worse surgical outcomes. Therefore, the proposed strategy can be used for noninvasive preoperative MVI diagnosis and to assess postoperative recurrence and survival risks for patients with HCC, which is valuable for tailoring individualized treatment strategies—for example, identifying candidates who may benefit from adjuvant therapy or intensified postoperative surveillance.

This study had limitations. First, it was a retrospective study, and prospective study samples should be included for further evaluation in the future. Second, although power analysis was performed, larger study samples are still needed in future research to validate the effectiveness and robustness of the proposed strategy. Third, we did not include radiologic features to identify risk factors for MVI and subsequently construct a combined model. The model’s performance may be further improved by incorporating radiologic features in future studies. Clinical variables were not incorporated into the predictive models in our study, as this was a data-driven decision. However, in future studies involving independent cohorts, this choice may not be applicable if certain clinical variables are found to be significantly associated with the target variable. Fourth, potential bias factors in SHAP analysis included the sample size, data distribution, model complexity, and feature scaling. In practical applications, these factors should be carefully considered in a comprehensive manner. Fifth, survival analysis was performed only with the data from institution 1, as survival data from the other institutions were unavailable, which may have limited the generalizability of our findings based on survival analysis. Sixth, univariable analysis was used as the second step of feature selection to identify features that showed significant differences between groups. However, this approach may overlook nonlinear yet informative features, potentially impairing the model performance and increasing the risk of overfitting. Seventh, the workflow can be completed within 10 minutes per case on a regular consumer-grade PC, with the main time limitation stemming from manual segmentation. Future integration of automated segmentation techniques could potentially reduce the processing time to just a few seconds.

In conclusion, we evaluated a habitat-based strategy to quantitatively encode the ITH and PTH of HCC, enabling noninvasive and accurate diagnosis of MVI and prognostic risk stratification. Consequently, this approach holds promise for guiding clinical decision-making by accurately identifying MVI and assessing postoperative recurrence risk at an early stage, aiding clinicians in formulating optimal treatment plans, and ultimately providing clinical benefits to patients.
